# Investigation on Rheological Properties and Storage Stability of Modified Asphalt Based on the Grafting Activation of Crumb Rubber

**DOI:** 10.3390/polym11101563

**Published:** 2019-09-25

**Authors:** Juan Xie, Yueming Yang, Songtao Lv, Yongning Zhang, Xuan Zhu, Cece Zheng

**Affiliations:** 1 School of Traffic and Transportation Engineering, Changsha University of Science and Technology, Changsha 410114, China; 17101030028@stu.csust.edu.cn (Y.Y.); lst@csust.edu.cn (S.L.); zhangyongning0424@163.com (Y.Z.); cslgzx@stu.csust.edu.cn (X.Z.); zcccslgdx@163.com (C.Z.); 2 National Engineering Laboratory for Highway Maintenance Technology, Changsha University of Science and Technology, Changsha 410114, China

**Keywords:** acrylamide, chemical grafting, modified asphalt, rheology, storage stability, compatibility, chemical cross-linking

## Abstract

Acrylamide with a double bond and amide group can not only copolymerize with macromolecules of crumb rubber but also react with acidic groups in asphalt, so it was selected as a modifier to activate crumb rubber through chemical graft action. The purpose is to improve the compatibility between crumb rubber and asphalt and thus improve the rheological properties and storage stability of rubber asphalt. Infrared spectroscopy (IR) and scanning electron microscopy (SEM) were used to characterize the crumb rubbers and their modified asphalt. It was found that the crumb rubber of grafting acrylamide had better compatibility in asphalt due to its larger specific surface area and chemical reaction with asphalt. In addition, the high temperature rheological test, low temperature creep test, and polymer separation test were carried out to study the effect of grafted activated crumb rubber on the properties of modified asphalt. The results showed that compared with modified asphalt with common crumb rubber (CRMA), the rheological properties and storage stability of modified asphalt with grafting activated crumb rubber (A–G–R) were improved significantly. The results of microscopic and macroscopic tests show that the activated rubber particles have a larger contact area with asphalt due to a rougher surface and the chemical cross-linking between rubber particles and asphalt further strengthens their interaction. Therefore, there is a relatively stable blend system formed in modified asphalt, and its performance of modified asphalt has been improved.

## 1. Introduction

Waste tires will become solid waste after aging, mechanical wear, and scrapping. China’s scrap tire production has ranked first in the world for ten consecutive years and is expected to reach 20 million tons by 2020 [1]. Traditional treatment methods such as incineration, landfill or simple accumulation will not only occupy land resources, but also release harmful substances, pollute soil and groundwater, which will endanger human survival [2,3]. Rubber waste containing macromolecule elastomers can be ground into crumb rubber and added to asphalt, which can not only improve the road performance of asphalt and driving comfort, but also improve the economic and environmental benefits [4,5]. Meanwhile, as a main material used in pavement, traditional asphalt is facing severe challenges due to growing traffic volume and changing climate [6,7,8]. Therefore, a lot of modified asphalts are prepared to meet the modern transportation requirements for asphalt materials [9]. Among of them, crumb rubber modified asphalt has attracted wide attention because of good comprehensive performance. However, due to the poor compatibility between crumb rubber and base asphalt, the separation of crumb rubber modified asphalt is very easy to occurs during storage, and the poor storage stability is usually considered as the main factor limiting its application and development [10,11,12,13].

High sedimentation rate and the separation of rubber particles generate at high temperature because of low compatibility, which results in poor storage stability of modified asphalt [14,15]. The main reason is that crumb rubber is made by mechanical grinding from waste tires, which contains vulcanized cross-linking structure, and the surface of crumb rubber is inert. Besides, crumb rubber has great difference with asphalt in molecular structure and polarity, resulting in low compatibility between crumb rubber and asphalt [16]. Therefore, the recently research work focuses on improving the storage stability of rubber modified asphalt without sacrificing comprehensive performance, among which compound modification and rubber powder activation are currently the most common methods.

The performance of rubber modified asphalt can be improved by adding admixtures. As a commonly used asphalt modifier, styrene-butadiene-styrene (SBS) was added to rubber modified asphalt at high temperature and improved its processability and storage stability [17]. Yu et al. [18] found that after being incorporated into rubber modified asphalt through high-speed shearing, the layered nano-montmorillonite transformed to intercalated or exfoliated structures, enhancing storage stability. Xue et al. [19] wrapped rubber modified asphalt with steel slag powder by granulation machine and prepared compound modified asphalt, and the results showed the softening point differences decreased to 2.1 °C. Compound modification can improve the storage stability of rubber modified asphalt, but the reactions among different modifiers are complicated and are not conducive to mechanism analysis.

Activation of crumb rubber by physical or chemical approaches has been proved to be effective in improving the compatibility between crumb rubber and asphalt [20]. Yu et al. [21] demonstrated that microwave radiation could destroy the surface of vulcanization network of rubber particles and improve the surface activity of rubber so as to improve the viscoelasticity and storage stability of modified asphalt. Deep oxidation of rubber powder can reduce the cross-linking structure between rubber particles, consequently improving the fluidity of rubber asphalt [22,23]. Li et al. [24] treated crumb rubber with hydrogen peroxide and found that the obtained rubber had more dense voids and larger specific surface area, which improved the interaction between rubber and asphalt. Shatanawi et al. [25] found that the crumb rubber activated by solution of furfural (C_5_H_4_O_2_) had higher activity and enhanced compatibility with asphalt, and the modified asphalt had improved rutting resistance and storage stability. The activation of crumb rubber has received increasing attention due to the following advantages: the activation method and activator can be selected in accordance with the demand application, the modification mechanism is simple, the improvement effect is obvious.

Crumb rubber is inert due to its vulcanized crosslinked structure and thus has relatively low binding ability with asphalt [26]. But present studies indicate crumb rubber are rich in unsaturated doubles, which are easily broken at high temperature and react with C=C bonds in acrylamide to form long chains [27]. The iodine values measured through Wijs method by Jin et al. also showed that there were 0.153 moles double bonds per 100 g rubber powder, which ensured the graft reaction of acrylamide and crumb rubber proceeded smoothly [28]. Moreover, there are acid groups in asphalt [29], which can react chemically with the alkaline amide groups in acrylamide [30]. Therefore, crumb rubber activated by acrylamide through chemical grafting method can increase the surface alkaline composition of rubber, which enhances the bonding ability between rubber and base asphalt, and then improves the compatibility between rubber powder and asphalt. Hence, both the chemical and physical interaction between crumb rubber and asphalt can be improved through the activation of rubber with acrylamide, and rubber modified asphalt with good storage stability can be prepared.

In this paper, the crumb rubber was graft activated by acrylamide in accordance with the principle of molecule design. Infrared spectroscopy (IR) and scanning electron microscopy (SEM) were used to characterize the chemical structure and morphology of common and activated rubber and the modified asphalt, respectively. The Brookfield viscosity test, rheological tests of modified asphalts were carried out. In order to study the effect of activated crumb rubber on the rheological properties of modified asphalt at high and low temperatures, the 175 °C viscosity, complex modulus (G*), phase angle (*δ*), rutting factor (G*/sinδ), creep stiffness modulus (*S*), and creep rate (m) of modified asphalt with common crumb rubber (CRMA) and modified asphalt with grafting activated crumb rubber (A–G–R) were compared. In addition, the softening point difference and rutting factor separation index (SI) were used to characterize the storage stability of modified asphalt. The results of macro- and micro- tests show that the better compatibility and stronger interaction between activated crumb rubber (AM–CR) and asphalt can effectively improve the performance of rubber asphalt and increase its feasibility in application.

## 2. Experimental

### 2.1. Materials

Base asphalt binder of grade 70 supplied by Maoming Xinlu Building Materials Company (Maoming, China) and crumb rubber of 80 mesh provided from Dujiangyan Huayi Rubber Company (Chengdu, China) were used, and the technical parameters are shown in Table 1 and Table 2, respectively. Acrylamide was used as the grafting monomer and potassium persulfate was used as the initiator, which were both of analytical grade and obtained by Shenzhen Jitian Chemical Co., Ltd. (Shenzhen, China).

### 2.2. The Activation of Crumb Rubber

Waste rubber contains additives such as antioxidants and plasticizers [31], which will prevent the graft reaction. Therefore, acetone was used to remove impurities on the surface of crumb rubber at first. Then the surface grafting activation of rubber was carried out, and the specific steps were as follows: Firstly, 2.5 g potassium persulfate, 40 g acrylamide, and 100 g rubber powder were mixed into 1000 mL ultrapure water. Then the mixed solution was stirred by magnetic force stirrer for 240 min at 80 °C. Among them, potassium persulfate was used as the initiator to graft acrylamide monomer onto rubber particles. Finally, after being washed by a large amount of ultra-pure water and dried in an oven, the activate crumb rubber (AM–CR) was obtained.

### 2.3. Preparation of Modified Asphalt

In this study, the untreated crumb rubber (CR) and AM–CR were added into the base asphalt with 10%, 15%, 20%, and 25%, respectively, and mixed by high-speed shear instrument. The shear time is 60 min and the shear temperature is 180 °C. Finally, the modified asphalts were put into the oven at 180 °C and swelled for 60 min. To ensure comparability of performance, the reference base asphalt was also treated through the same process.

### 2.4. Test Methods

The infrared absorption spectrum of rubbers and their modified asphalt were obtained by using the Nicolet is10 Fourier transform infrared spectrometer (Thermo Fisher, Waltham, MA, USA). The spectrum ranged from 4000 to 500 cm^−1^, and scanning was repeated 32 times.

The morphology of rubbers and their modified asphalts was observed by using the S-3000N scanning electron microscope produced by Hitachi Company (Tokyo, Japan), with magnification of 200 and 500 times. Samples were pretreated by gold sputtering prior to the observation.

According to JTG-T0625-011 test method, the Brookfield viscometer was used to test the viscosity of modified asphalt. The rotation rate was 100 r/min, the rotor type was 27, and the test temperature was 175 °C.

The complex shear modulus, phase angle and rutting factor of modified asphalt was measured in accordance with JTG-T0628-2011 by a dynamic shear rheometer (DSR, MCR 302, Antongpa, Graz, Austria). The rheological tests are carried out by parallel plates with a diameter of 25 mm and a spacing of 1 mm under strain controlling mode (temperatures range from 58 to 88 °C, *ω* = 10 rad/s).

The rectangular asphalt samples (125 mm × 12.7 mm × 6.35 mm) were prepared and the low temperature creep tests were conducted in accordance with JTG-T0627-2011 by a bending beam rheometer (BBR, TE-BBR, Cannon, Tokyo, Japan). The load was 0.98 N and continued for 4 min; the creep stiffness modulus S and creep rate m value were measured at loading time 1 min.

Modified asphalt was injected into standard aluminium tube (diameter 25 mm, height 140 mm). After sealing, the modified asphalt was placed vertically in the oven at 163 °C for 12, 24, 48, 60, and 72 h. Then the aluminium tube was taken out and frozen for 240 min in −4 °C and divided into 3 sections to measure the softening point and rheological properties of the upper and lower sections. The softening point difference and rutting factor separation index (SI) of the upper and lower sections of tubes were characterized for storage stability of modified asphalt in this paper.

## 3. Results and Discussion

### 3.1. Microscopic Characterization of Crumb Rubber

**Figure 1:**
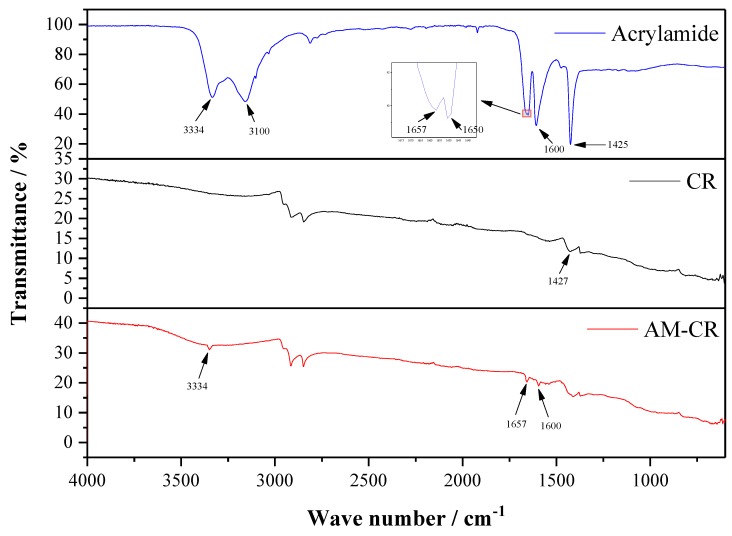
Infrared absorption spectrums of crumb rubber (CR) and activated crumb rubber (AM–CR).

Infrared spectroscopy (IR) is widely used in qualitative and quantitative analysis of organic compounds, especially in the analysis of chemical structure of polymers. In this paper, IR was used to characterize the chemical structure of crumb rubber. The infrared absorption spectrums of CR and AM–CR are shown in Figure 1. It can be seen that there were three new absorption peaks in the infrared absorption spectrums of AM–CR compared with that of CR, including the absorption peak at 3334 cm^−1^ caused by the stretching vibration of –NH_2_ (primary amide) [32], the absorption peak at 1657 cm^−1^ and 1600 cm^−1^ caused by the stretching vibration of –NHCO– [3], while these new chemical bonds are derived from acrylamide. In the infrared absorption spectrum of acrylamide, the peaks appearing at 3100 cm^−1^ and 1650 cm^−1^ were the stretching vibration peaks of =CH_2_ and C=C [33], respectively, which did not appear in the infrared absorption spectrum of AM–CR, and the absorption peak at 1425 cm^−1^ caused by the stretching vibration of –C–N overlaps with the absorption peak at 1427 cm^−1^ caused by anti-symmetric angular vibration of –CH_3_ in rubber. The results show that acrylamide was grafted onto rubber particles.

In the process of grafting, potassium persulfate acts as initiator and decomposes into sulfate ion (SO_4_^2−^) at high temperature to form a redox system. The allyl at the end of macromolecular chain of rubber has higher activity at high temperature, which makes the C=C bond on allyl easy to break and polymerize with the C=C bond of acrylamide to form long chains. The specific reaction process is as follows:

Production of primary radicals:

$$S_{2}O_{8}^{2-}\to 2SO_{4}^{2-}$$

$$SO_{4}^{2-}+-CH_{2}C(CH_{3})=CHCH_{2}CH_{2}-\to HSO_{4}^{-}+-CH_{2}C(CH_{3})=CHC(H)CH_{2}-$$

Generation of monomer radicals:

$$-CH_{2}C(CH_{3})=CHC(H)\cdot CH_{2}-+Q\to -CH_{2}C(CH_{3})=CHC(H)Q\cdot CH_{2}-$$

Graft reaction (chain growth):

$$-CH_{2}C(CH_{3})=CHC(H)Q_{n}\cdot CH_{2}-+Q\to -CH_{2}C(CH_{3})=CHC(H)Q_{n+1}\cdot CH_{2}-$$

Termination:

$$-CH_{2}C(CH_{3})=CHC(H)Q_{n+1}\cdot CH_{2}-+-CH_{2}C(CH_{3})=CHC(H)Q_{n+1}\cdot CH_{2}-\to$$

$$\begin{array}{r} -CH_{2}C(CH_{3})=CHC(H)\underset{|}{Q_{n+1}}CH_{2}-\\ -CH_{2}C(CH_{3})=CHC(H)\underset{}{Q_{n+1}}CH_{2}- \end{array}$$

where *Q* is the graft monomer acrylamide ($CH_{2}=CHCONH_{2}$).

**Figure 2:**
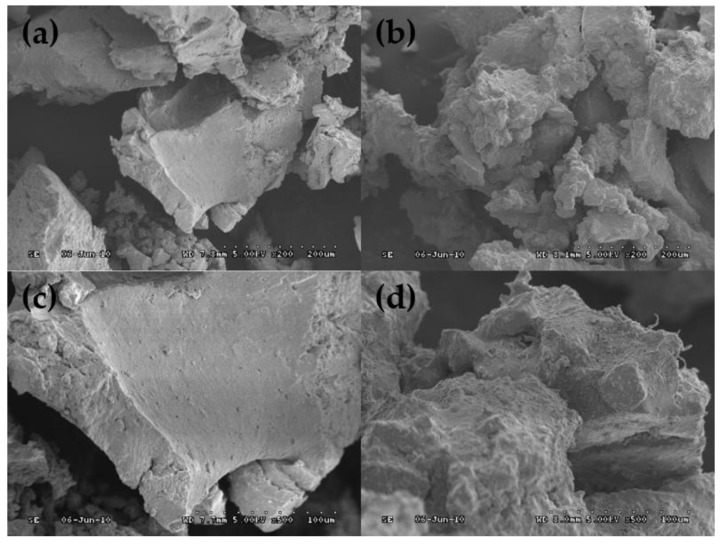
Scanning electron microscopy (SEM) images of CR and AM–CR. (**a**) The image of CR (200×); (**b**) The image of AM–CR (200×); (**c**) The image of CR (500×); (**d**) The image of AM–CR (500×).

Scanning electron microscopy was used to observe the morphology of the common rubber and the activated rubber. The images are shown in Figure 2. It can be seen that the surface of crumb rubber (CR) is very smooth and flat. This structure is not conducive to the dispersion of crumb rubber in asphalt binder, nor can it make crumb rubber bond well with asphalt [34,35]. However, the acrylamide grafted rubber powder (AM–CR) has a rough and uneven surface with larger specific surface area, which can increase the contact area between rubber particles and asphalt and enhance the swelling effect of rubber in asphalt [36]. Therefore, the rubber can absorb more light components in asphalt in the swelling process.

### 3.2. Characterization of Chemical Structure

**Figure 3:**
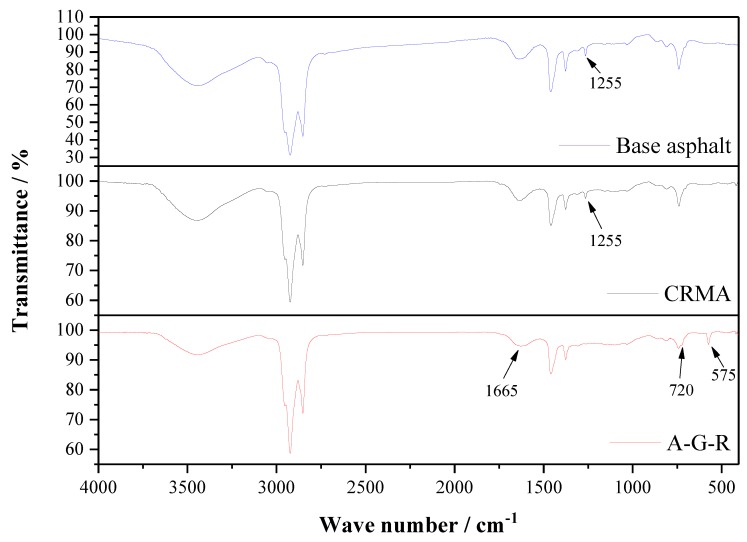
Infrared absorption spectrums of CRMA and A–G–R.

**Figure 4:**
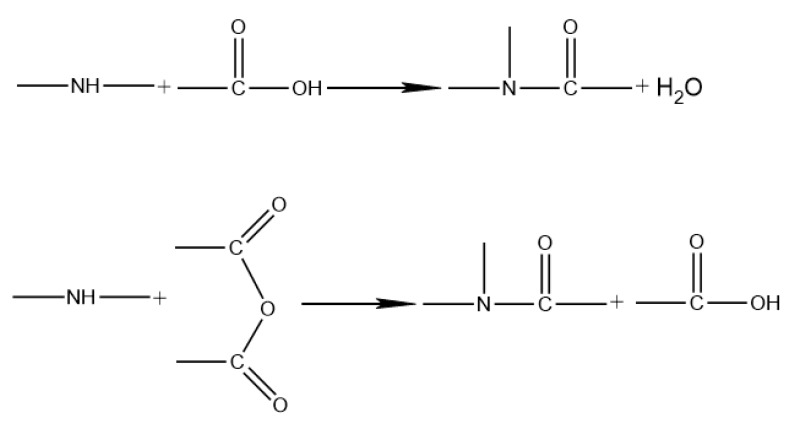
The reaction between activated rubber and asphalt.

Infrared absorption spectrums of base asphalt and asphalt modified with 20% wt of CR and AM–CR are shown in Figure 3. According to Figure 1, the infrared absorption peaks of CR mainly concentrate in the range of 2700–3000 cm^−1^ and 1200–1500 cm^−1^, which are similar to base asphalt (Figure 3) and concealed by base asphalt. There is no new peak found in the curve of CRMA, so the addition of CR to asphalt belongs to physical blending. In the curve of A–G–R, the absorption peak at 575 cm^−1^ belongs to bending vibration of N–C=O (common amide). The peak at 720 cm^−1^ originated from bending vibration of O–C=O and the peak of 1665 cm^−1^ is originated from stretching vibration C=O, which both belong to –COOH [33]. It is noteworthy that there is a peak at 1225 cm^−1^ originating from anhydride in both spectrums of base asphalt and CRMA. The content of anhydride in asphalt is low [37] and increases during high temperature due to the oxidation of carboxylic acid [38]. However, this peak disappears in the spectrum of A–G–R, which is probably because anhydride groups are very reactive and consumed by amide groups. According to the FTIR analysis for the chemical functional groups of base asphalt and modified asphalt, a reaction between amide groups in grafted rubber and acid groups in asphalt. The reaction equation is listed below (Figure 4). It shows that not only physical blending, but also chemical reaction, occurs between activated rubber and asphalt.

### 3.3. High Temperature Rheological Properties

**Figure 5:**
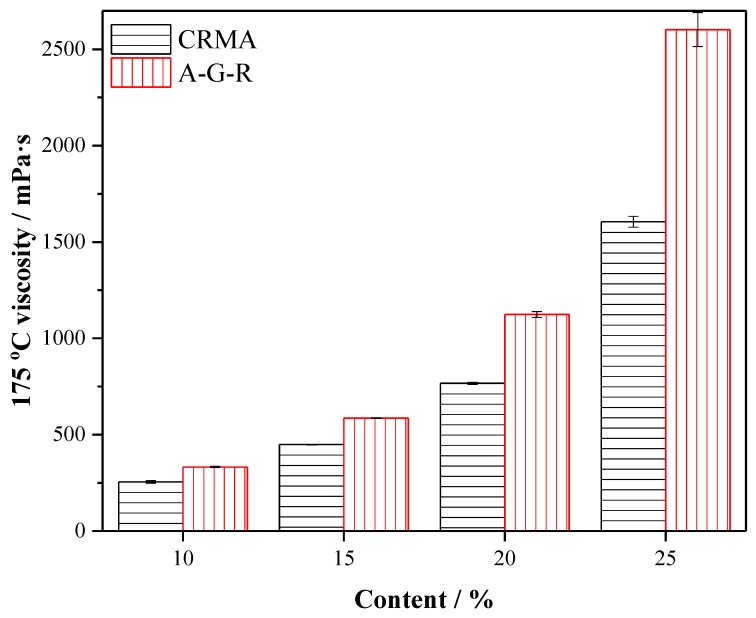
The 175 °C viscosity of CRMA and A–G–R.

In this part, the variation trend of 175 °C viscosity, complex modulus (G*), phase angle (*δ*), and rutting factor (G*/sinδ) with the increase of rubber content was analyzed to study the effect of activated rubber on the high temperature rheological properties of modified asphalt. Viscosity, as an important property of asphalt binder, represents the pumping capacity and workability of binder at high temperature. Figure 5 shows the 175 °C viscosity of modified asphalt at different contents. It can be seen that the viscosity of modified asphalt increases with the increase of rubber content. When the rubber content exceeds 20% wt, the increase range of viscosity increases greatly. This is because the viscosity of rubber asphalt is determined by the viscosity of asphalt binder, the interaction between rubber and asphalt, and the particle spatial effect of granular rubber particles [39,40]. The increase of rubber can improve the interaction and spatial effect, so the viscosity of modified asphalt is increased. However, when the rubber content reaches a certain level, there are not enough oil contents in asphalt to make the rubber powder swell completely [39]. The rubber particles after absorbing component may contact each other more easily because of expansion. Friction between the rubber particles limits the fluidity of the system and makes the viscosity increase sharply. At the same content, the viscosity of A–G–R is higher than that of CRMA, and with the increase of content, the growth rate is also increasing. This is because the chemical interaction between the amide group grafted on the molecular chain of rubber particles and the acid group in asphalt strengthens the cross-linking between them, consequently enhancing the interfacial viscous effect between rubber and asphalt, so A–G–R has higher viscosity.

**Figure 6:**
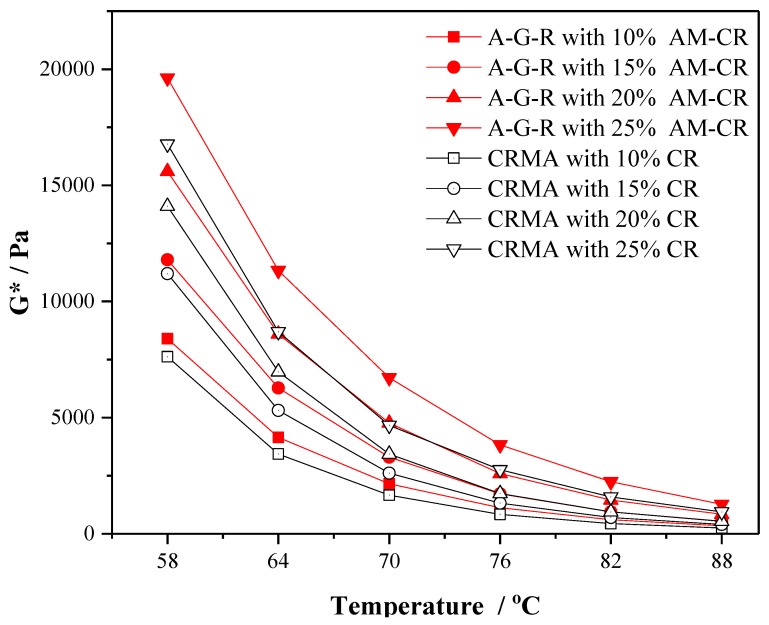
The complex modulus (G*) of CRMA and A–G–R.

**Figure 7:**
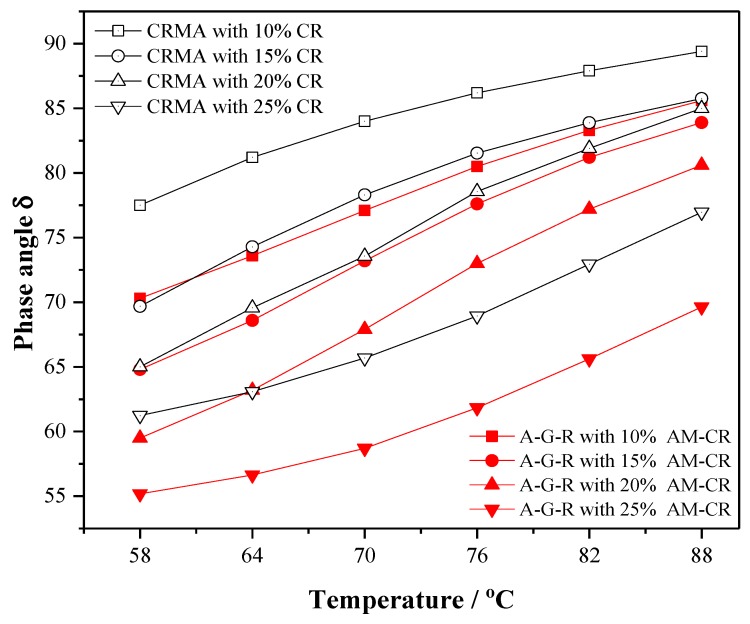
The phase angle (*δ*) of CRMA and A–G–R.

Complex modulus (G*) and phase angle (*δ*), as important rheological parameters at high temperature, can effectively reflect the deformation resistance and viscoelasticity of asphalt binder. Figure 6 and Figure 7 show the trend of complex modulus (G*) and phase angle (*δ*) of CRMA and A–G–R with temperature at different contents, respectively, to study the effect of AM–CR on the rheological property of rubberized asphalt at high temperature.

As shown in Figure 6, with the increase of rubber content, the G* of modified asphalts are increasing, which means that the increase of both rubber contents can effectively improve the deformation resistance of modified asphalt under dynamic loading. Compared with CRMA, at the same temperature, the G* of A–G–R increases significantly at any content, which indicates that A–G–R has greater deformation resistance under dynamic loading. The increase of G* in rubberized asphalt is attributed to the decrease of weight fraction of light components in modified asphalt during the swelling of rubber [41]. AM–CR with larger specific surface area has better combination with asphalt, so it can absorb more light components in asphalt during the swelling process.

As shown in Figure 7, the phase angle δ of the rubber asphalts decreases with the increase of the content, which shows that the increasing rubber can improve the elasticity of the binder. At the same temperature, the phase angle of A–G–R is lower than that of CRMA at any content. This may be because that the chemical interaction between activated rubber and asphalt enhances the three-dimensional network structure. Besides, AM–CR retains the inherent elastic nature of rubber and provides additional elasticity for the whole system. Hence the elastic response of A–G–R increases, and its deformation recovery ability improves.

**Figure 8:**
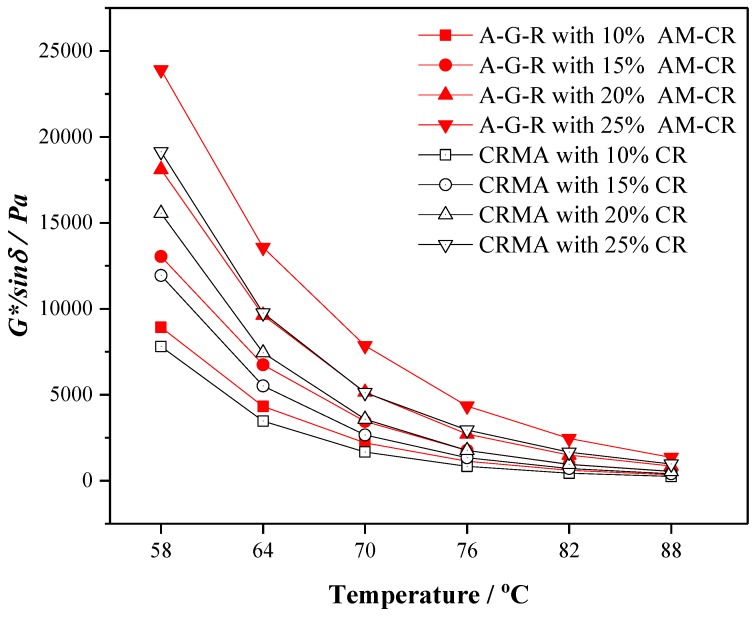
The rutting factor (G*/sinδ) of CRMA and A–G–R.

The rutting factor (G*/sinδ) belongs to the rheological property of asphalt, which characterizes the anti-rutting ability of asphalt. The larger the G*/sinδ value is, the better the anti-rutting performance of asphalt is. Figure 8 shows the variation trend of G*/sinδ of CRMA and A–G–R as a function of temperature. The G*/sinδ of modified asphalt decreases with the increase of temperature, which indicates that the higher the temperature is resulted in the greater the possibility of rutting. The rutting factor of modified asphalt improved with the increase of rubber content at the same temperature, which is due to the high elasticity of rubber itself and a stable network structure formed by the swelling of rubber. However, the rutting factor of A–G–R is larger than that of CRMA at any temperature, and the gap between them increases with the increase of the content. This means that A–G–R has better rutting resistance, and the higher the content of AM–CR is, the more significant the impact of AM–CR on the rutting resistance of modified asphalt is, which is attributed to the chemical crosslinking between AM–CR and asphalt. Meanwhile, compared with hydrogen peroxide activated crumb rubber [16], acrylamide grafted crumb rubber has a better effect on the high temperature performance of modified asphalt.

### 3.4. Low Temperature Creep Properties

The creep stiffness modulus *S* and creep rate *m* obtained by bending beam rheometer test are used to characterize the tenacity and stress relaxation ability of modified asphalt at low temperature, which can directly reflect the low temperature crack resistance of modified asphalt. Table 3 shows the stiffness modulus *S* and creep rate *m* of CRMA and A–G–R at −12 °C, −18 °C, and −24 °C. When the temperature decreases, the *S* value of modified asphalt increases and the m value decreases, which means that the modified asphalt has a greater possibility of cracking. At the same temperature, the *S* value of the modified asphalts decreases, and the *m* value of the modified asphalts increases with the increase of the content, which indicates that the low temperature cracking resistance of the modified asphalts improves with the increase of rubber content. This is mainly due to the fact that rubber powder, as an elastic material, has less stiffness at low temperatures and can increase overall tenacity [41,42]. And crumb rubber with increasing content could continuously absorb the light components in base asphalt, thus increasing the proportion of asphaltene, and the network structure between asphalt and rubber powder is formed, which further enhances the tenacity of rubberized asphalt. At the same content, A–G–R has a smaller *S* value and larger *m* value. This is because in addition to the elasticity of rubber itself, the interaction between asphalt and rubber is also an important factor determining the low temperature performance of rubber asphalt [43,44]. AM–CR can produce chemical cross-linking with asphalt, which strengthens the interaction between asphalt and rubber by forming a three-dimensional network structure. Moreover, a larger specific surface area can provide a larger area for chemical reaction, which further constitutes a relatively stable three-dimensional network structure. Therefore, A–G–R exhibits better cryogenic performance.

### 3.5. Morphology

**Figure 9:**
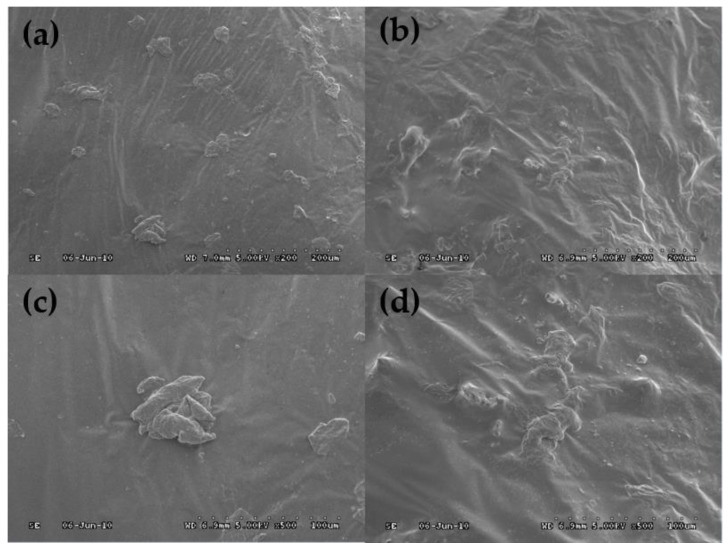
SEM images of CRMA and A–G–R. (**a**) The image of CRMA (200×); (**b**) The image of A–G–R (200×); (**c**) The image of CRMA (500×); (**d**) The image of A–G–R (500×).

The compatibility between rubber particles and asphalt affects not only the morphology and structure of modified asphalt, but also its performance. Figure 9 shows the morphology of CRMA and A–G–R. It shows the low swelling degree of crumb rubber in common crumb rubber modified asphalt, the agglomeration between rubber particles, and the clear interface with asphalt, which indicates that the compatibility between rubber and base asphalt is poor [17]. However, activated crumb rubber dissolves and swells sufficiently in base asphalt, without obvious agglomeration, and its interface with base asphalt is blurred, which indicates that the compatibility between crumb rubber and base asphalt is improved significantly after activation treatment. This is due to the presence of basic amino groups in the acrylamide molecular chain grafted on the surface of rubber. Hence, AM–CR has a strong interaction with acid groups in asphalt and hinders the phase separation between rubber powder and base asphalt, which improves their compatibility, and thus forms a relatively stable blending system.

### 3.6. Storage Stability

**Figure 10:**
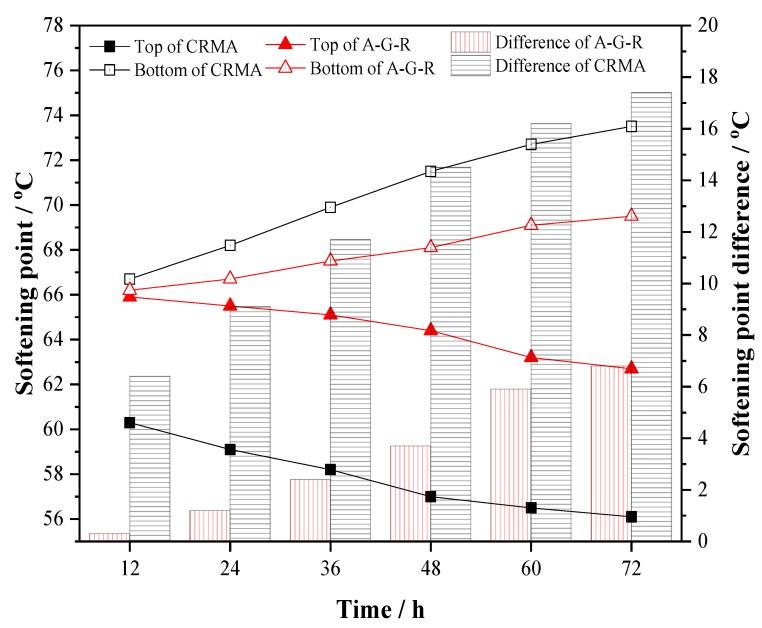
The softening point and softening point difference of CRMA and A–G–R.

Softening point difference is often used to evaluate the thermal storage stability of modified asphalt. The smaller the softening point difference is, the closer the performance of upper and lower parts of modified asphalt is, and the better the storage stability is [45]. Asphalt modified with 20% of CR and AM–CR were stored for 12 h, 24 h, 36 h, 48 h, 60 h, and 72 h at 163 °C to simulate the separation state in practical application. The results of softening point and softening point difference are shown in Figure 10, where the broken line diagram represents the softening point of modified asphalt at the top and bottom of tube, and the column diagram represents the difference between them. It can be seen from the figure that the softening point difference of both samples (with CR and AM–CR) increases with the extension of storage time, which indicates that there is separation between modifiers and asphalt in the storage of two kinds of modified bitumen at high temperature. However, compared with CRMA, the softening point difference of A–G–R decreases significantly in each storage period. After 72 h storage, the softening point difference of A–G–R was 6.8, while that of CRMA was 17.4. The softening point difference of A–G–R was reduced by 60%, indicating that activated rubber could effectively improve the storage stability of rubber asphalt. This is attributed to the strong interaction and good compatibility between AM–CR and asphalt. Especially in the first 12 h, the softening point difference of A–G–R is only 0.3, and it was believed that there is almost no separation. This means that in practical application, it does not need to be continuously stirred in a shorter transportation distance, which can reduce additional energy consumption and save costs.

**Figure 11:**
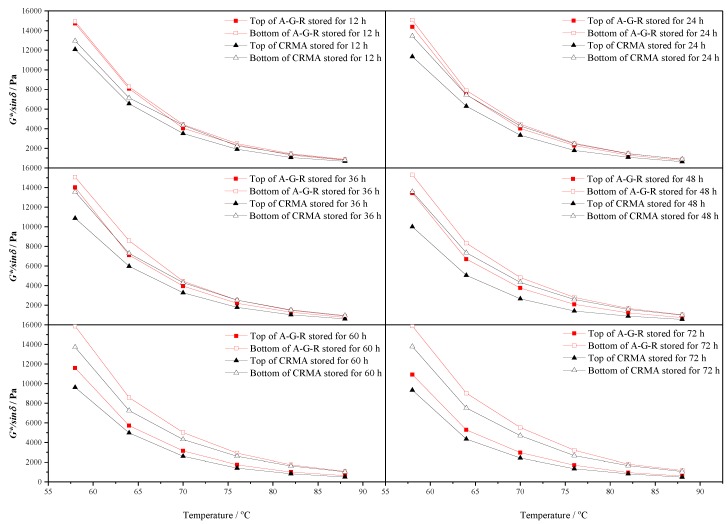
The rutting factor-temperature curves of CRMA and A–G–R.

For further studying the effect of AM–CR on the storage stability of rubber asphalt, the rutting factors of modified asphalt at the upper and lower parts of the aluminum tube were also measured. Figure 11 shows that the temperature dependence curve of rutting factor of top and bottom of CRMA and A–G–R with 20%wt content stored at 163 °C for 12 h, 24 h, 36 h, 48 h, 60 h, and 72 h. It reflects the rheological properties of the upper and lower parts of modified asphalt after high temperature storage. It can be seen that the rutting factor decreases with the increase of temperature, but the G*/sinδ of the bottom of the modified asphalt is always higher than that of the top, and the difference of the rutting factor between the top and the bottom of the modified asphalt increases with the prolongation of storage time. This shows that after a period of thermal storage, the separation of modified asphalt occurs, and the degree of separation of modifier and asphalt increases with the prolongation of storage time.

Considering the difference between CRMA and A–G–R in high temperature performance, direct comparison of their rutting factors can not reflect the gap of their separation degree. Therefore, the rutting factor separation index is introduced to evaluate the storage stability of modified asphalt in this study. Separation index (*SI*) [46] is defined as the rutting factor ratio of modified asphalt at the bottom and top of tube as follows:

*SI* = (G*/sinδ)*_bottom_*/(G*/sinδ)*_top_*, (1)

where (G*/sinδ)*_bottom_* is the rutting factor of polymer modified asphalt at the lower part of the aluminum tube, and (G*/sinδ)*_top_* is the rutting factor of polymer modified asphalt at the upper part of the aluminum tube. The higher the SI value is, the greater the separation degree of polymer modified asphalt is.

**Figure 12:**
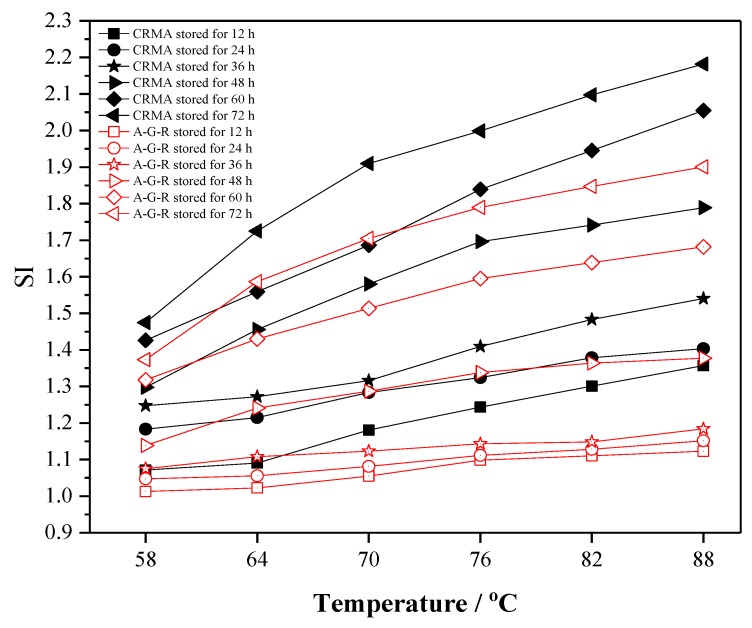
The separation index (*SI*) curves of CRMA and A–G–R.

Figure 12 shows the change trend of the rutting factor separation index (SI) of the upper and lower parts for rubberized asphalt (20%wt content of crumb rubber) after being stored at 163 °C for 12 h, 24 h, 36 h, 48 h, 60 h, and 72 h. The *SI* value of two kinds of modified asphalt increases with the increase of temperature, and the initial value and growth rate of SI value increase with the extension of storage time, which shows the poor storage performance of rubberized asphalt. However, the SI value of A–G–R is always lower than that of CRMA, which indicates that AM–CR is more compatible with asphalt than CR and can effectively improve the storage stability of rubberized asphalt. When the storage time is 12 h, the initial and final SI values of A–G–R are 1.01 and 1.12 respectively, indicating much small separation degree of A–G–R stored in a short time, which is consistent with the results of softening point difference.

**Figure 13:**
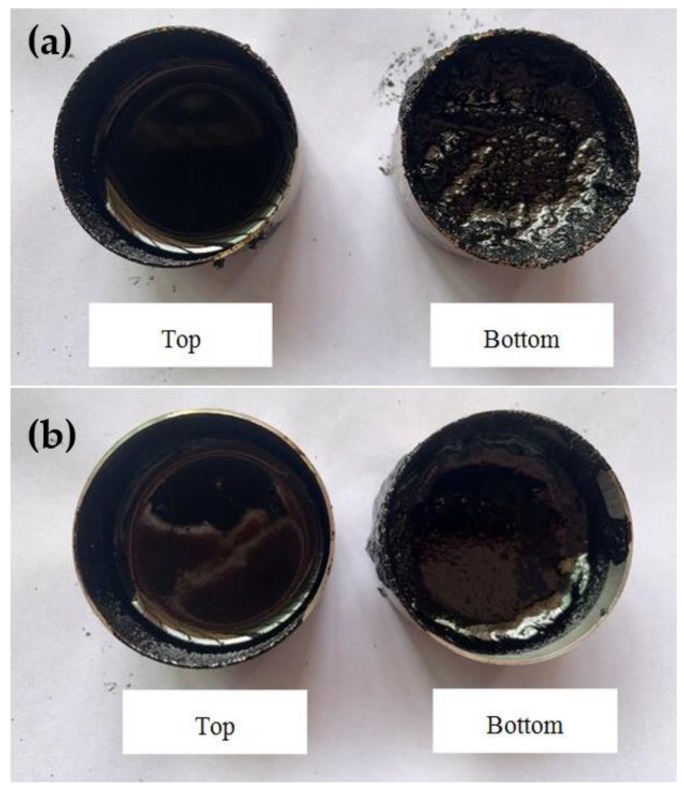
A–G–R and CRMA stored 72 h at 163 °C. (**a**) The image of CRMA; (**b**) the image of A–G–R.

In addition, the upper and lower parts of CRMA and A–G–R after being stored at 163 °C for 72 h are poured into the aluminum box, respectively, as shown in Figure 13. It can be observed by the naked eye that in CRMA much crumb rubber settles to the lower part and agglomerates, resulting in obvious phase separation. On the contrary, the problem of separation between AM–CR and asphalt is alleviated obviously.

It is generally believed that the reason that rubber asphalt cannot form a relatively stable system is that only physical interaction between rubber and asphalt takes place [20,47,48]. The chemical cross-linking between AM–CR with asphalt is an important reason for the good storage stability of A–G–R. In the A–G–R, the cross-linking structure between AM–CR and asphalt formed by chemical bonds and the cross-linking structure formed by swelling between AM–CRs constitute a relatively stable three-dimensional network structure. Therefore, the mixed system shows a relatively stable state.

## 4. Conclusions

In this paper, acrylamide, which can not only copolymerize with macromolecules on the rubber, but also react with acidic groups in asphalt, is used as graft modifier to activate crumb rubber. The microcosmic tests of rubbers and modified asphalts included infrared absorption spectrum analysis and scanning electron microscopy. And for studying the effect of activated crumb rubber on the properties of modified asphalt, the high temperature rheological index, the low temperature creep index, and the storage stability index were measured. The following conclusions can be drawn:

(1) Infrared absorption spectrum showed that the new absorption peaks in AM–CR were originated from acrylamide, and the C=C bond in acrylamide disappeared due to polymerization grafting. The grafting activation of rubber particles is a chemical reaction process. And compared with CRMA, the absorption peaks of A–G–R produced by –C–O–C– disappeared at 1255 cm^−1^, but there were three absorption peaks on the infrared absorption spectrum, which confirmed that not only physical blending but also chemical reaction took place between AM–CR and asphalt.

(2) Scanning electron microscopy (SEM) images show that the surface of activated rubber particle is rougher, and its specific surface area is larger, which is conducive to its combination with asphalt. And the interface between AM–CR and asphalt is blurred, which shows that they have good compatibility.

(3) The results of 175 °C viscosity, complex modulus, phase angle, and rutting factor show that the activated crumb rubber can make modified asphalt have better deformation resistance and recovery ability under dynamic load, consequently improving the high temperature rheological properties of modified asphalt.

(4) The results of stiffness modulus and creep rate at different temperatures show that the activated crumb rubber can improve the tenacity and stress relaxation ability of modified asphalt at low temperature, and thus improving its creep properties of modified asphalt at low temperature.

(5) The good compatibility and the strong interaction of activated crumb rubber with asphalt due to a larger contact area chemical cross-linking make A–G–R exhibit good storage stability in different storage periods.

## Figures and Tables

**Table 1:** Properties of base asphalt binder.

Item	Units	Test Results	Standard
Penetration (25 °C, 100 g, 5 s)	0.1 mm	64.0	JTG-T0604-2011
Softening temperature	°C	48.1	JTG-T0606-2011
Ductility (15 °C, 5 cm/min)	cm	>100	JTG-T0605-2011
Kinematic viscosity (135 °C)	mPa s	158.5	JTG-T0625-2011
Density	g/cm^3^	1.034	JTG-T0603-2011
RTFO treated at 163 °C for 85 min			
Quality change	%	−0.061	JTG-T0610-1-2011
Residual penetration ratio (25 °C)	%	80.0	JTG-T0610-2-2011
Residual ductility (5 °C)	cm	7.0	JTG-T0605-2011

**Table 2:** Properties of crumb rubber.

Item	Result	Standard
Water content (%)	0.96	HG/TXXX-2001 7.2.2
Ash content (%)	9.3	GB4498
Acetone extract content (%)	13.6	GB/T3516
Density (g/cm^3^)	1.05	GB/T533
Tensile strength (MPa)	6.4	GB/T528
Elongation at break (%)	855	GB/T52

**Table 3:** Creep stiffness modulus *S* and creep rate *m* value of CRMA and A–G–R.

Simple	−12 °C		−18 °C		−24 °C	
	*S*	*m*	*S*	*m*	*S*	*m*
CRMA with 10% CR	115.0	0.368	231.3	0.324	457.7	0.275
CRMA with 15% CR	82.3	0.389	182.3	0.333	386.0	0.282
CRMA with 20% CR	75.6	0.396	159.7	0.350	297.3	0.296
CRMA with 25% CR	45.2	0.398	70.4	0.357	196.0	0.308
A–G–R with 10% AM–CR	104.0	0.371	219.7	0.329	411.0	0.282
A–G–R with 15% AM–CR	72.5	0.383	178.3	0.341	344.3	0.295
A–G–R with 20% AM–CR	67.3	0.413	147.7	0.359	240.0	0.307
A–G–R with 25% AM–CR	33.5	0.422	63.4	0.368	143.3	0.309
